# Boosting Photocatalytic Performance of ZnO via Ce‐Induced d‐Band Modulation: Synthesis and Rhodamine B Degradation

**DOI:** 10.1002/open.70260

**Published:** 2026-07-15

**Authors:** Bingwei Zhong, Bo Xia, Nan Wang, Jing Hu, Xulei Zhao

**Affiliations:** ^1^ College of Jiyang Zhejiang A&F University Zhuji China; ^2^ School of Earth Atmosphere and Environment Monash University Clayton Victoria Australia

**Keywords:** CeO_2_/ZnO heterojunction, magnetically recoverable catalysts, reactive oxygen species, ZnO‐based photocatalysts

## Abstract

Synthetic dyes are persistent organic pollutants in aquatic environments, posing serious ecological and health risks. Here, we report a morphology‐guided and interface‐engineered ZnO‐based photocatalyst for efficient dye degradation. Among morphology‐controlled ZnO architectures, sphere‐like ZnO exhibited superior activity owing to favorable structural and surface characteristics. Subsequent deposition of CeO_2_ formed a CeO_2_/ZnO heterojunction, in which mixed‐valence Ce^3+^/Ce^4+^ species promoted interfacial charge redistribution and reactive oxygen species generation, leading to an enhancement exceeding 50% in degradation kinetics compared with pristine ZnO. Density functional theory calculations revealed that interfacial electron transfer induces an upward shift of the Zn d‐band center, facilitating oxygen activation. The catalytic performance was further optimized under representative reaction conditions, and a magnetically separable CeO_2_/ZnO–ZnFe_2_O_4_ composite was constructed to enable efficient catalyst recovery and long‐term stability. This work demonstrates how morphology control and heterojunction design cooperatively regulate electronic structure and photocatalytic reactivity in oxide‐based systems for environmental remediation.

## Graphical abstract

Morphology‐guided CeO_2_/ZnO heterojunctions enhance photocatalytic dye degradation with over 50% faster degradation kinetics through interfacial charge redistribution and promoted reactive oxygen species generation. DFT and EPR reveal how electronic structure modulation facilitates oxygen activation, providing mechanistic insights for designing efficient and recyclable ZnO‐based photocatalysts.

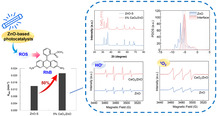

## 1 Introduction

With the continued expansion of industrial activities, water pollution has emerged as an increasingly critical environmental concern. Effluents released from textile and dyeing industries contain substantial amounts of unabsorbed organic dyes [1, 3]. Owing to their chemically stable structures, many of these dyes are highly resistant to natural degradation. Furthermore, numerous dyes have been reported to exhibit carcinogenic, teratogenic, and mutagenic effects [4, 5], thereby posing significant risks to both ecosystems and human health. Consequently, it is crucial to develop efficient, cost‐effective, and environmentally benign methods for the treatment of dye‐laden wastewater.

Traditional treatments for the removal of dyes include physical, chemical (coagulation or adsorption), and biological processes, yet these processes are of low efficiency or high operational cost. As a result, semiconductor‐based photocatalysis has gained widespread attention as an effective and promising alternative for dye degradation [6, 7]. During the photocatalytic process, the interaction between the semiconductor material and the incident photons excites electrons (e^‒^), resulting in the formation of electron–hole pairs (e^‒^/h^+^) and other reactive oxygen species (ROS), which immediately initiate subsequent photoredox reactions [8, 9]. Metal oxide semiconductors, such as TiO_2_, ZnO, Fe_2_O_3_, and NiO‐based materials, have been extensively explored for dye removal owing to their tunable band structures and efficient utilization of photogenerated charge carriers [10, 11]. For example, ZnO nanoparticles have been successfully applied for the degradation of various organic dyes in aqueous systems, highlighting their potential for wastewater treatment applications [12]. In addition, modified metal oxide systems, such as Ni/NiO‐based composites, have also been developed to enhance dye removal performance by improving charge transfer processes and surface reaction sites [13].

In particular, zinc oxide (ZnO), a direct wide‐bandgap (3.37 eV) n‐type semiconductor, has emerged as a leading candidate because of its high exciton binding energy (60 meV), nontoxic nature, low cost, and strong photocatalytic activity [14, 16]. Although titanium dioxide (TiO_2_) remains one of the most extensively explored photocatalysts [17, 18], its application is limited by narrow UV absorption and relatively low quantum efficiency [19, 20]. In contrast, ZnO absorbs a broader range of UV light and often exhibits higher quantum efficiency, resulting in superior photocatalytic performance for organic pollutant degradation. Moreover, ZnO displays strong visible‐light emission, which can be quantitatively quenched by h^+^ scavengers. This property has enabled the simultaneous detection and degradation of harmful electron‐donating chemicals, such as chlorophenols, by monitoring emission quenching [21, 22]. Despite these advantages, the practical utilization of ZnO is still hindered by two intrinsic limitations: its wide bandgap, which restricts photoresponse to UV light (λ < 385 nm, ~4% of the solar spectrum), and the rapid recombination of photogenerated e^‒^ and h^+^ [23, 25].

Various strategies have been proposed to improve the photocatalytic activity of ZnO, including size adjustment, loading with metal or nonmetal atoms, noble metal deposition, modification of electronic and optical properties, heterojunction construction, and coupling with carbon materials [26, 29]. Compared to bulk ZnO, micro/nanoscale ZnO offers a significantly larger specific surface area, which improves dye adsorption and thus enhances photocatalytic performance. In addition, micro/nanoscale ZnO exhibits diverse morphologies, such as particles, rods, sheets, and spheres, that further influence their catalytic behavior [30, 31]. Beyond size and morphology engineering, metal loading is another effective means to improve ZnO photocatalysis [32]. Among various elements, rare‐earth ions have attracted particular interest due to their unique 4f electron configuration [33, 36], which enables them to act as electron traps and suppress the recombination of photogenerated e^‒^/h^+^ [37, 38]. Rare‐earth ions incorporation may also induce a redshift in the bandgap transition of ZnO, thereby improving their visible‐light response [39]. Among rare‐earth elements, cerium (Ce) is especially promising because of its high abundance and strong capability to modulate bandgap structure and charge‐carrier dynamics. Ce loading has been shown to markedly enhance ZnO photocatalysis; for example, Sin et al. reported a 1.9‐fold activity improvement for ZnO loaded with 1.5 at% Ce, attributing the enhancement to suppressed e^‒^‐h^+^ recombination [40]. However, the amount of Ce incorporated is crucial, as excessive loading can impair photocatalytic performance, as demonstrated by Chang et al. [41] for Ce‐loaded ZnO nanorods.

The primary objective of this work is to establish a rational strategy for enhancing ZnO‐based photocatalysts by systematically linking structural architecture with interfacial electronic regulation. To this end, ZnO materials with distinct morphologies were first comparatively evaluated to identify an optimal structural platform for photocatalytic reactions. On this basis, CeO_2_ was deposited onto the ZnO surface to construct a well‐defined CeO_2_/ZnO heterojunction, enabling interfacial charge redistribution and enhanced oxygen activation through mixed‐valence cerium species. In parallel, key reaction parameters were optimized to maximize catalytic efficiency under representative conditions. Furthermore, to address catalyst recovery and long‐term operational stability, a magnetically separable CeO_2_/ZnO–ZnFe_2_O_4_ composite was designed. Through the integration of morphology control, heterojunction engineering, and functional composite construction, this study provides mechanistic insight into structure‐interface‐reactivity relationships in oxide photocatalysts and offers a general design framework for efficient and recyclable systems for environmental remediation.

## 2 Materials and Methods

### 2.1 Materials

Zinc nitrate (Zn(NO_3_)_2_·6H_2_O, ≥99%), hexamethylenetetramine (HMTA, ≥99%), rhodamine B (RhB, ≥99%), hydrazine hydrate (N_2_H_4_·H_2_O, 97%), zinc acetate dehydrate (Zn(CH_3_COO)_2_·2H_2_O, ≥99%), 2‐methylimidazole (≥99%) and cerium nitrate hexahydrate (Ce(NO_3_)_3_⋅6H_2_O, ≥99%) were supplied by Sigma–Aldrich (St. Louis, MO, USA). Hydrogen peroxide (H_2_O_2_, 30%) solution, trisodium citrate (98%), ammonia (NH_3_·H_2_O, 25%) solution, ethanol, hydrochloric acid (HCl), sodium hydroxide (NaOH) and ferric chloride (FeCl_3_, 97%) were obtained from Sinopharm Chemical Reagent Co., Ltd. (Shanghai, China). Other chemicals utilized for the synthesis of ZnO‐based materials, at least of reagent grade, were used as received without purification. All solutions were prepared by Milli‐Q water (18 MΩ·cm) obtained from a Stakpure OmniaTap water purification system (Perculiar Instrument Technology, UK) and used within a week.

### 2.2 Synthesis of ZnO‐Based Materials

To elucidate structure–performance relationships and improve the efficiency of photocatalysts, a series of ZnO‐based materials were synthesized, including zeolitic imidazolate framework (ZIF)‐derived ZnO, Ce‐loaded ZnO with various morphologies, and magnetic CeO_2_/ZnO composites.

#### 2.2.1 Synthesis of ZnO

To evaluate the influence of morphology on photocatalytic activity, ZnO nanostructures with rod‐like, platelet‐like, and sphere‐like morphologies were synthesized, while porous ZIF‐derived ZnO was prepared as a reference sample. Each sample was synthesized at least three times under identical conditions to confirm the reproducibility of the morphology.

##### 2.2.1.1 Rod‐Like ZnO (ZnO‐R)

ZnO‐R was synthesized by a hydrothermal precipitation method. Briefly, 0.2745 g of Zn(CH_3_COO)_2_·2H_2_O and 1.2000 g NaOH were accurately weighed and separately dissolved in 15 and 30 mL of deionized water, respectively. After complete dissolution, the Zn(CH_3_COO)_2_ solution was slowly added dropwise into the NaOH solution under continuous magnetic stirring. The resulting suspension was stirred at 400 rpm for 30 min to ensure sufficient mixing and homogeneous nucleation. Subsequently, the obtained precursor solution was transferred into a 50 mL Teflon‐lined stainless steel autoclave and hydrothermally treated at 200 °C for 5 h in a muffle furnace. After the reaction, the autoclave was naturally cooled to room temperature. The resulting white precipitate was collected by centrifugation, washed several times with deionized water and ethanol to remove residual ions and impurities, and dried in a vacuum oven at 80 °C for 8 h.

##### 2.2.1.2 Platelet‐Like ZnO (ZnO‐P)

ZnO‐P was prepared following the same procedure as the ZnO‐R, except that 0.1830 g Zn(CH_3_COO)_2_·2H_2_O and 0.8000 g NaOH were used, and the hydrothermal time was extended to 30 h to promote lateral crystal growth. The resulting product was washed and dried under identical conditions.

##### 2.2.1.3 Sphere‐Like ZnO (ZnO‐S)

ZnO‐S was synthesized by dissolving 1.2 mmol Zn(NO_3_)_2_ · 6H_2_O, 1.2 mmol HMTA, and 0.3 mmol trisodium citrate in 80 mL deionized water. The resulting solution was sealed in a 100 mL Teflon‐lined stainless steel autoclave and heated at 90 °C for 4 h. After cooling to room temperature, the precipitate was collected by centrifugation, washed with deionized water and ethanol, and dried at 60 °C for 12 h. The obtained precursor was calcined in air at 500 °C for 2 h, followed by natural cooling to room temperature.

##### 2.2.1.4 Porous ZIF‐Derived ZnO (ZIF‐ZnO)

ZIFs, a subclass of metal–organic frameworks composed of metal ions bridged by imidazolate ligands, exhibit high porosity, excellent thermal stability, and tunable composition. In particular, ZIF‐8 features a uniform polyhedral morphology and a high zinc content, making it an ideal sacrificial template for producing porous ZnO with well‐controlled structure and crystallinity [20].

To synthesize the ZIF‐ZnO, ZIF‐8 was first prepared. Briefly, 0.8782 g Zn(CH_3_COO)_2_·2H_2_O was dissolved in 4.773 g deionized water under stirring, while 0.6572 g 2‐methylimidazole was dissolved in 8.7043 g ammonia solution (25 wt%). The Zn(CH_3_COO)_2_ solution was then added dropwise into the 2‐methylimidazole solution under continuous stirring. After mixing, the suspension was stirred at room temperature for 10 min to complete the coordination reaction. The obtained precipitate was collected by centrifugation, washed with deionized water until neutral, and dried at 60 °C overnight to obtain ZIF‐8. Subsequently, the dried ZIF‐8 powder was calcined in a tube furnace under an air atmosphere at 400 °C for 3 h to remove organic ligands and obtain porous ZIF‐ZnO.

#### 2.2.2 Synthesis of Ce‐Loaded ZnO Nanospheres (CeO_2_/ZnO)

To enhance the charge separation efficiency and improve photocatalytic performance, cerium was introduced into ZnO material through controlled loading. After the selection of the ideal ZnO‐S material by characterization, Ce‐loaded ZnO was synthesized under the same conditions as unloaded materials, except for the addition of different amounts of Ce(NO_3_)_3_·6H_2_O (0.012–0.084 mmol) to achieve Ce loading levels of 1, 3, 5, and 7 mol%, labeled as x% CeO_2_/ZnO.

#### 2.2.3 Synthesis of Magnetic CeO_2_/ZnO Nanospheres (M‐CeO_2_/ZnO)

To enable efficient catalyst recovery while maintaining photocatalytic stability, ZnFe_2_O_4_ was incorporated as a magnetic component into the CeO_2_/ZnO system. ZnFe_2_O_4_ possesses a chemically robust spinel structure and exhibits good stability under aqueous and photo‐irradiated conditions [42]. Moreover, the presence of Zn^2+^ in its lattice ensures good chemical compatibility with the ZnO matrix, allowing stable interfacial integration without significantly perturbing the host crystal structure [43].

ZnFe_2_O_4_ was synthesized via a hydrothermal reduction method. Briefly, 2 mmol Zn(NO_3_)_2_ · 6H_2_O and 4 mmol FeCl_3_ · 6H_2_O were dispersed in 60 mL deionized water under magnetic stirring at room temperature. Then, 8 mL of 5 mol/L hydrazine hydrate was added dropwise, and the mixture was stirred for 30 min. The resulting suspension was transferred into a 100 mL Teflon‐lined autoclave and heated at 180 °C for 14 h. After cooling to room temperature, the product was collected, washed with deionized water and ethanol, and dried at 60 °C. The M–CeO_2_/ZnO composite was prepared by introducing 10 wt% ZnFe_2_O_4_ into the 5% CeO_2_/ZnO precursor solution, followed by hydrothermal treatment under the same hydrothermal conditions used for CeO_2_/ZnO synthesis.

### 2.3 Characterization of ZnO‐Based Materials

The synthesized materials were characterized by powder X‐ray diffraction (XRD) using a PANalytical X’Pert^3^ diffractometer. The surface morphology and microstructure were observed by scanning electron microscopy (SEM) using a JEOL JEM‐2100F field‐emission electron microscope. Elemental composition was determined by energy‐dispersive X‐ray spectroscopy (EDS) attached to the SEM. Specific surface area of the samples was analyzed by nitrogen adsorption–desorption isotherms using the Brunauer–Emmett–Teller (BET) method on a Micromeritics ASAP 2460 instrument. X‐ray photoelectron spectroscopy (XPS) was performed on a Thermo Scientific instrument equipped with an Al Kα X‐ray source to analyze the surface chemical composition and oxidation states. UV–vis absorption and diffuse reflection spectra (DRS) of the photocatalysts were recorded on a UV–vis spectrophotometer (Shimadzu UV‐3600i Plus) to estimate the optical bandgap energy. Electron paramagnetic resonance (EPR) spectra were collected at room temperature on a Bruker EMXplus spectrometer to identify and quantify ROS during the photocatalytic processes. The Ce leaching after photocatalytic cycling was determined using inductively coupled plasma optical emission spectroscopy (ICP‐OES, Agilent 5110).

### 2.4 Density Function Theory (DFT) Calculations

Spin‐polarized DFT calculations were performed with the Perdew–Burke–Ernzerhof (PBE) exchange‐correlation functional in a plane‐wave pseudopotential implementation using the Vienna ab initio simulation package (VASP). A cutoff energy of 450 eV for the plane‐wave basis set was employed. The method of Methfessel–Paxton (MP) with a smearing width of 0.20 eV was adopted for transition metal surfaces and interfaces. The DFT + U formalism was used to describe the localized (strongly correlated) 4f electrons in Ce, and UCe ‐ JCe = 4.5 eV was used, which has been successfully used in previous works to describe supported Ce‐based species on transition metal surfaces. A (3 × 3 × 1) Gamma‐centered k‐point sampling was adopted with a 15 Å vacuum to separate the slab perpendicular to the surface. The convergence criteria for the residual force and energy during structure relaxation were set to 0.05 eV/Å and 1 × 10−5 eV, respectively.

### 2.5 Photocatalytic Performance

RhB was selected as a representative cationic dye pollutant because it is widely used as a benchmark molecule in photocatalytic studies, facilitates comparison with reported ZnO‐ and CeO_2_‒based photocatalysts, and is suitable for establishing structure–activity relationships in adsorption‐assisted photocatalytic degradation. RhB exhibits a maximum absorption at 554 nm, and its absorbance is directly related to its concentration according to the Lambert–Beer law (Equation (1)). Therefore, the concentration change of RhB can be evaluated by recording the temporal evolution of its characteristic absorption peak at 554 nm using UV–vis spectroscopy.

(1) A=εbc

Where *A*is the absorbance of RhB at 554 nm, *ε* is the molar absorption coefficient of RhB (M^‒^^1^ cm^‒^^1^), *b* is the penetration depth of light (cm), and *c* is the concentration of RhB (M).

Consequently, the photocatalytic degradation efficiency (η) of RhB can be calculated by the following equation:

(2) $$\eta =\frac{A_{0}-A_{t}}{A_{0}}$$

Where A_0_ and A_t_ are the RhB absorbance before and after UV irradiation, respectively.

In a detailed procedure, the catalyst (10, 20, 30, 40 mg·L^‒^^1^) and RhB (1, 3, 5, 7 mg·L^−1^) were fully dispersed in a beaker using ultrasound. The system was then allowed to reach adsorption–desorption equilibrium over a period of 15 min. Subsequently, the UV lamp was placed at a fixed distance of 10, 15, 20 cm above the reaction solution under 365 nm irradiation. The incident photon flux (I_0_, 9.0 × 10^‒^^8^ Einstein·cm^‒^^2^·s^‒^^1^) was determined by PNA (7.9 μM)/pyr (1.24 mM) chemical actinometry (Text S1) [44].

Sample aliquots were withdrawn at preset time points (−15, 0, 15, 30, 60, 90, and 120 min). The point at −15 min denotes the initial state immediately after mixing all components, while 0 min corresponds to the onset of irradiation following a 15 min dark equilibration period. The remaining time points represent the irradiation time. After centrifugating the extracted reaction solution, the supernatant was collected, and its absorbance was measured using UV spectroscopy to calculate the RhB degradation rate. Parallel samples without the catalyst or 365 nm UV light irradiation were run concurrently.

## 3 Results and Discussion

### 3.1 Characterization of ZnO Materials

**FIGURE 1:**
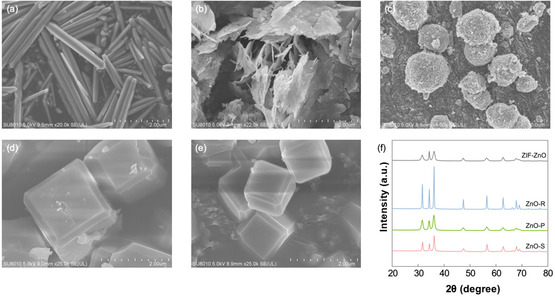
SEM images of ZnO samples with different morphologies: (a) rod‐like ZnO (ZnO‐R), (b) platelet‐like ZnO (ZnO‐P), (c) sphere‐like ZnO (ZnO‐S), (d) ZIF‐derived ZnO (ZIF‐ZnO), and (e) ZIF‐8. (f) XRD patterns of the four samples, confirming the hexagonal wurtzite structure of ZnO and the distinct crystallinity originating from the different synthesis routes.

SEM imaging revealed distinct morphologies for the four ZnO samples (Figure 1). The hydrothermally synthesized rod‐like ZnO (ZnO‐R) consisted of well‐defined hexagonal rods with high aspect ratios and uniform dispersion, confirming successful directional crystal growth (Figure 1a). The platelet‐like ZnO (ZnO‐P) exhibited thin, sheet‐like structures with lateral extension and frequent stacking, characteristic of layered crystal growth (Figure 1b). Moreover, the sphere‐like ZnO (ZnO‐S) showed uniformly dispersed spherical particles with rough surface textures and diameters (Figure 1c). In contrast, the ZIF‐derived ZnO (ZIF‐ZnO) retained a polyhedral morphology inherited from the ZIF‐8 precursor and displayed a porous surface framework, consistent with the decomposition of the organic template during calcination (Figure 1d,e).

XRD analysis was conducted to examine the crystal structures of the morphology‐controlled ZnO samples (ZnO‐R, ZnO‐P, ZnO‐S) and the ZIF‐derived porous ZnO (ZIF‐ZnO) (Figure 1f). The three hydrothermally synthesized ZnO samples exhibited sharp and well‐defined diffraction peaks matching the hexagonal wurtzite ZnO phase (JCPDS 36‐1451). The intense and narrow peaks indicated high crystallinity, with all three materials showing strong reflections. In contrast, the ZIF‐derived ZnO exhibits noticeably broader and weaker diffraction peaks, reflecting a lower degree of crystallinity. This difference in crystallinity can be attributed to their distinct formation mechanisms. The higher crystallinity of hydrothermally synthesized ZnO can be attributed to the relatively controllable aqueous environment, which facilitates gradual nucleation and the oriented growth of well‐ordered wurtzite‐type ZnO crystals. In contrast, ZIF‐ZnO is obtained through the thermal decomposition of ZIF‐8 precursors, during which the rapid removal of organic ligands induces partial collapse of the original framework, followed by incomplete reconstruction of the Zn‐O lattice. Although the diffraction peak positions of ZIF‐ZnO are consistent with the wurtzite ZnO phase, the significantly broadened and weakened peaks indicate reduced crystallinity and limited crystallite growth. This phenomenon is mainly associated with the preservation of a porous framework and structural rearrangement during calcination, which hinder the formation of long‐range ordered ZnO domains. In addition, residual carbonaceous species and lattice distortions generated during pyrolysis may further increase structural disorder, resulting in a ZIF‐ZnO structure with lower crystallinity.

### 3.2 Performance Evaluation of ZnO and CeO_2_/ZnO Materials

#### 3.2.1 Performance‐Based Screening of ZnO Supports

**FIGURE 2:**
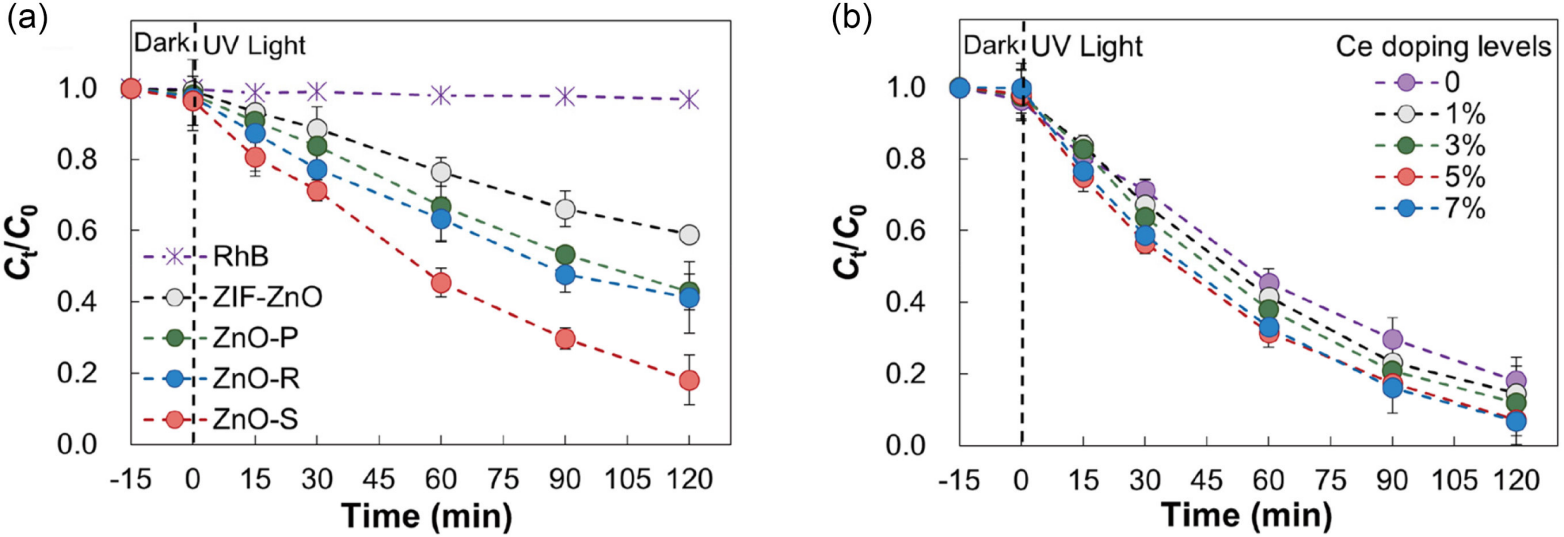
The degradation efficiency of RhB by different ZnO materials (a), and different Ce loading levels on ZnO‐S (b). Reaction conditions: RhB 5 mg/L, catalyst dosage 20 mg/L, irradiation distance 15 cm, LDE lamp (365 nm, 15w), pH 7.0, 25 °C. Error bars represent the standard deviation of 2 replicates.

The photocatalytic performance of different ZnO materials was assessed through the degradation of RhB under 365 nm UV irradiation, with the reaction progress monitored by absorbance changes at 554 nm. A negligible degradation of RhB was observed under UV irradiation without catalyst, suggesting that direct photolysis contributed little to RhB removal. Notably, ZnO‐S exhibited the highest photocatalytic activity among all ZnO variants, achieving 81.9% RhB degradation within 120 min under UV irradiation (Figure 2a). The highest photocatalytic activity of ZnO‐S is likely related to its spherical architecture, which promotes multiple internal reflections of incident light, thereby improving light‐harvesting efficiency. This effect increases the probability of photon absorption within the structure, enabling more effective excitation of charge carriers under UV irradiation [45, 46]. In contrast, ZnO‐P and ZnO‐R showed moderate performance, whereas ZIF‐ZnO exhibited the lowest degradation efficiency. This difference can be mainly attributed to the lower crystallinity of ZIF‐ZnO. Highly crystalline ZnO synthesized via the hydrothermal route possesses an ordered wurtzite structure, which facilitates the transport of photogenerated carriers and reduces e^‒^/h^+^ recombination. In comparison, the lower crystallinity of ZIF‐ZnO introduces more structural disorder and defect‐related states, which may hinder efficient charge migration. Although appropriate defects can act as charge trapping sites, excessive or disordered defects may promote carrier recombination and reduce carrier utilization efficiency. Therefore, the inferior crystallinity and unfavorable defect distribution of ZIF‐ZnO contribute to its relatively poor photocatalytic performance.

#### 3.2.2 Enhanced Photodegradation Performance via Ce Loading

The photocatalytic activity of CeO_2_^‒^ modified ZnO‐S nanospheres exhibited a clear dependence on Ce loading, with the highest performance observed at 5 mol% Ce. As shown in Figure 2b, the photocatalytic activity increased with increasing Ce loading up to 5 mol% and subsequently decreased at higher loadings. A similar trend was observed at earlier time points (30 and 60 min), indicating a consistent enhancement effect across the entire reaction period. This trend can be attributed to the balance between the formation of effective CeO_2_/ZnO heterointerfaces and excessive surface modification. Moderate CeO_2_ deposition facilitated interfacial charge separation and provided additional active sites, thereby enhancing photocatalytic performance. In contrast, the 7 mol% CeO_2_/ZnO sample exhibited a significantly lower surface area (28.7 m^2^ g^−1^) than the 5 mol% sample (49.9 m^2^ g^−1^), as discussed in the BET analysis below, suggesting that excessive CeO_2_ deposition may partially cover ZnO surface sites and reduce the accessibility of reactive sites [47]. Moreover, excessive CeO_2_ loading could promote the aggregation of CeO_2_ domains and decrease the effective heterojunction contact area for charge transfer. The excessive defect states or interfacial recombination centers generated at high Ce contents may further accelerate charge carrier recombination, resulting in decreased photocatalytic activity. Therefore, an optimal Ce loading is essential to maximize the synergistic effects of interfacial charge transfer and surface catalytic reactions [48, 49].

According to the Lambert–Beer law, plots of [RhB]_0_/[RhB]_t_ versus t can be derived from the corresponding absorbance versus t data. Under the experimental conditions, results showed that the removal of RhB can be described using pseudo‐first‐order kinetics (R^2^ > 0.95) (Figure S1), as shown in Equations (3) and Equation (4) :

(3) $$\ln\;({\left[\mathrm{RhB}\right]}_{t}/{\left[\mathrm{RhB}\right]}_{0})=-k_{\mathrm{obs}}\cdot t$$

(4) $$\ln\;(A_{t}/A_{0})=-k_{\mathrm{obs}}\cdot t$$

where *k*_obs_ is the pseudo‐first‐order rate constant of RhB during the photocatalytic degradation process; [RhB]_0_ and [RhB]_t_ are the concentrations of RhB at time 0 and t, respectively; A_0_ and A_t_ are the absorbance of RhB at time 0 and t, respectively. Thus, the *k*_obs_ value for RhB degradation under the 5% CeO_2_/ZnO catalysis (0.0213 min^−1^) is higher than that under ZnO catalysis (0.014 min^−1^), demonstrating the superior catalytic activity of CeO_2_/ZnO. The *k*_obs_ values were determined from the slope of the linear fitting curves, while the small deviations of the intercepts from zero were attributed to experimental uncertainties and the adsorption–desorption equilibrium process before irradiation.

The photocatalytic performance of the CeO_2_/ZnO composite was further evaluated in comparison with previously reported catalysts (Table 1). Although the CeO_2_/ZnO system was synthesized through a relatively simple and scalable route, it achieved a high degradation efficiency of 92.7% within 120 min, outperforming or matching several catalysts prepared through more elaborate or multistep synthetic strategies. For instance, its activity exceeded that of Bi_2_WO_6_ and Sd‐TiO_2_ films, which required longer reaction times of 300 and 180 min, respectively. The performance of CeO_2_/ZnO was also comparable to that of more structurally engineered systems, such as NaBiS_2_/ZnO, Fe/g‐C_3_N_4_, and Z‐scheme g‐C_3_N_4_@Ag@Ag_3_PO_4_, despite the latter often relying on complex heterojunction construction or noble‐metal deposition. These comparisons highlight that the CeO_2_/ZnO composite offers a favorable balance between catalytic efficiency and synthetic simplicity, underscoring its practical potential for dye degradation applications.

**TABLE 1:** Comparison of the photocatalytic performance of CeO_2_/ZnO with reported ZnO‐based and composite photocatalysts.

Photocatalysts	Catalyst dosage, mg L^−1^	RhB concentration, mg L^−1^	Degradation, %	Reaction time, min	References
CeO_2_/ZnO	20	5	92.7	120	This study
Bi_2_WO_6_	1000	9.6	92	300	[50]
Sd‐TiO_2_ film	15	3.36	60	180	[51]
NaBiS_2_/ZnO	200	NS^a^	99.0	120	[52]
Fe/g‐C_3_N_4_	1000	20	99.5	120	[53]
Z‐scheme g‐C_3_N_4_@Ag@Ag_3_PO_4_	250	5	~98	60	[54]

a NS stands for not specified.

### 3.3 Characterization of CeO_2_/ZnO

**FIGURE 3:**
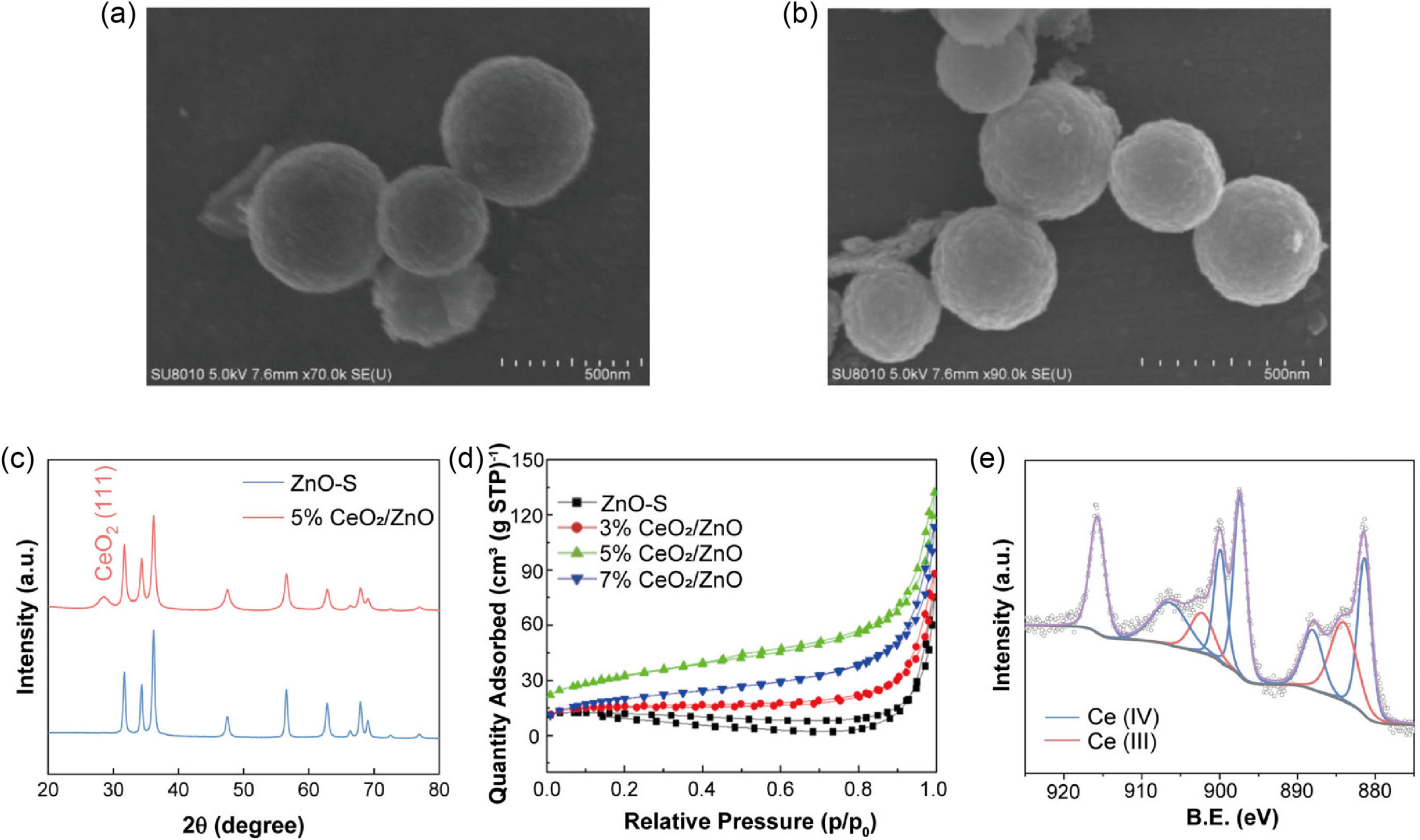
(a,b) SEM images of ZnO‐S and CeO_2_/ZnO. (c) XRD patterns of ZnO‐S and 5% CeO_2_/ZnO. (d) Nitrogen adsorption–desorption isotherms of ZnO‐S and Ce‐loaded samples. (e) High‐resolution Ce 3d XPS spectrum of 5% CeO_2_/ZnO.

SEM imaging was performed to examine the morphological features of the sphere‐like ZnO samples before and after Ce loading (Figure 3a,b). The pristine ZnO‐S consisted of well‐defined and uniformly distributed spherical particles with diameters of approximately 200–300 nm. After CeO_2_ deposition, the CeO_2_/ZnO composite retained the spherical morphology, but the particle surface became noticeably rougher, which can be attributed to the attachment of CeO_2_ nanoclusters.

To confirm the elemental composition of the samples, EDS analysis was performed on the Ce‐loaded ZnO spheres (Figure S2). The spectra showed clear signals corresponding to Zn, O, and Ce, with no additional elements detected within the instrument limits. This verifies that the composite consists solely of these three elements. The measured Ce content (~5.9 wt%) was close to the nominal loading of 5 wt%, indicating successful CeO_2_ deposition on the ZnO‐S surface. As expected, Zn and O accounted for the majority of the detected mass percentage due to the ZnO matrix.

The XRD patterns of ZnO‐S and Ce‐loaded ZnO‐S samples are presented in Figure 3c. Both materials exhibited diffraction peaks characteristic of the hexagonal wurtzite ZnO phase (JCPDS 36‐1451), indicating that Ce loading did not alter the crystal structure of ZnO. In the Ce‐loaded sample, an additional broad peak centered at 28.5° was observed, which corresponds to the (111) reflection of cubic CeO_2_ [55, 56]. This confirms the successful deposition of CeO_2_ on the ZnO‐S surface. No detectable shift in the ZnO diffraction peaks was observed, further indicating that Ce did not enter the ZnO lattice but instead formed a surface CeO_2_/ZnO heterojunction. Minor variations in peak intensity may arise from surface modification and light scattering differences introduced by CeO_2_ nanoclusters rather than changes in ZnO crystallinity.

Nitrogen adsorption–desorption measurements were carried out to evaluate the specific surface area and pore characteristics of the Ce‐loaded ZnO‐S samples (Figure 3d). All samples exhibited type IV isotherms with clear hysteresis loops, indicative of mesoporous structures. The specific surface areas of the materials ranged from 20 to 50 m^2^ g^−1^. Among them, the 5% CeO_2_/ZnO sample showed the highest surface area (49.9 m^2^ g^−1^), whereas the pristine ZnO‐S exhibited a lower value (26.9 m^2^ g^−1^). This trend is consistent with the increased surface roughness observed in the SEM images after CeO_2_ deposition. A moderate increase in surface area was observed at lower Ce loadings; however, the 7% CeO_2_/ZnO sample showed a decreased surface area (28.7 m^2^ g^−1^). This reduction may be attributed to excessive CeO_2_ depositing on the ZnO‐S surface, partially blocking accessible pores or reducing the effective exposed surface area. These results indicate that moderate Ce loading can enhance the textural properties of ZnO‐S, while excessive loading may lead to surface coverage and reduced porosity.

The chemical states of Ce in the CeO_2_/ZnO composite were examined by high‐resolution XPS of the Ce 3d region (Figure 3e). The Ce 3d spectrum exhibited the characteristic multiplet splitting of CeO_2_, which could be deconvoluted into several pairs of u (3d_3_/_2_) and v (3d_5_/_2_) components. The main doublets at higher binding energies were assigned to Ce^4+^ species, confirming the presence of a CeO_2_ phase on the ZnO‐S surface. In addition, weaker satellite peaks at lower binding energies were attributed to Ce^3+^ species, indicating that a fraction of Ce existed in a reduced state. The coexistence of Ce^4+^ and Ce^3+^ thus demonstrated that the CeO_2_ domains in the CeO_2_/ZnO composite possessed a mixed‐valence character, which is typically associated with oxygen‐vacancy formation and an enhanced redox capability. Such a Ce^3+^/Ce^4+^ redox couple is expected to facilitate interfacial charge transfer and contribute to the improved photocatalytic performance of the CeO_2_/ZnO sample.

### 3.4 Formation Mechanism of ZnO and CeO_2_/ZnO Heterostructures

Based on the above characterizations, the successful formation of CeO_2_/ZnO heterostructures is confirmed. Accordingly, a possible formation mechanism is proposed to illustrate the nucleation, growth, and interfacial assembly processes. Under hydrothermal conditions, HMTA gradually decomposes to generate OH^‐^, which drives the hydrolysis of Zn^2+^ and the formation of Zn(OH)_2_ nuclei, followed by dehydration to ZnO nanocrystals [57].

Simultaneously, Ce^3+^ species participate in competitive pathways depending on the local environment. Part of Ce^3+^ may interact with ZnO nuclei, inducing lattice distortion and defect formation, while the remaining species undergo hydrolysis and partial oxidation to CeO_2_ precursors within the reaction medium [58].

Sodium citrate acts as a complexing and structure‐directing agent, regulating metal ion hydrolysis and guiding the self‐assembly of ZnO primary nanocrystals into hierarchical microspheres via oriented attachment and surface‐energy minimization [59]. After calcination, the precursors are transformed into well‐crystallized ZnO microspheres decorated with CeO_2_ nanoparticles, forming intimate CeO_2_/ZnO heterointerfaces with abundant contact areas.

The resulting heterostructure facilitates interfacial charge transfer between CeO_2_ and ZnO, thereby suppressing electron–hole recombination. In addition, Ce‐induced defect states, particularly oxygen vacancies, further enhance charge separation and improve photocatalytic activity [60].

### 3.5 Optimization of Photocatalytic Conditions for RhB Degradation

#### 3.5.1 Catalyst Dosage

**FIGURE 4:**
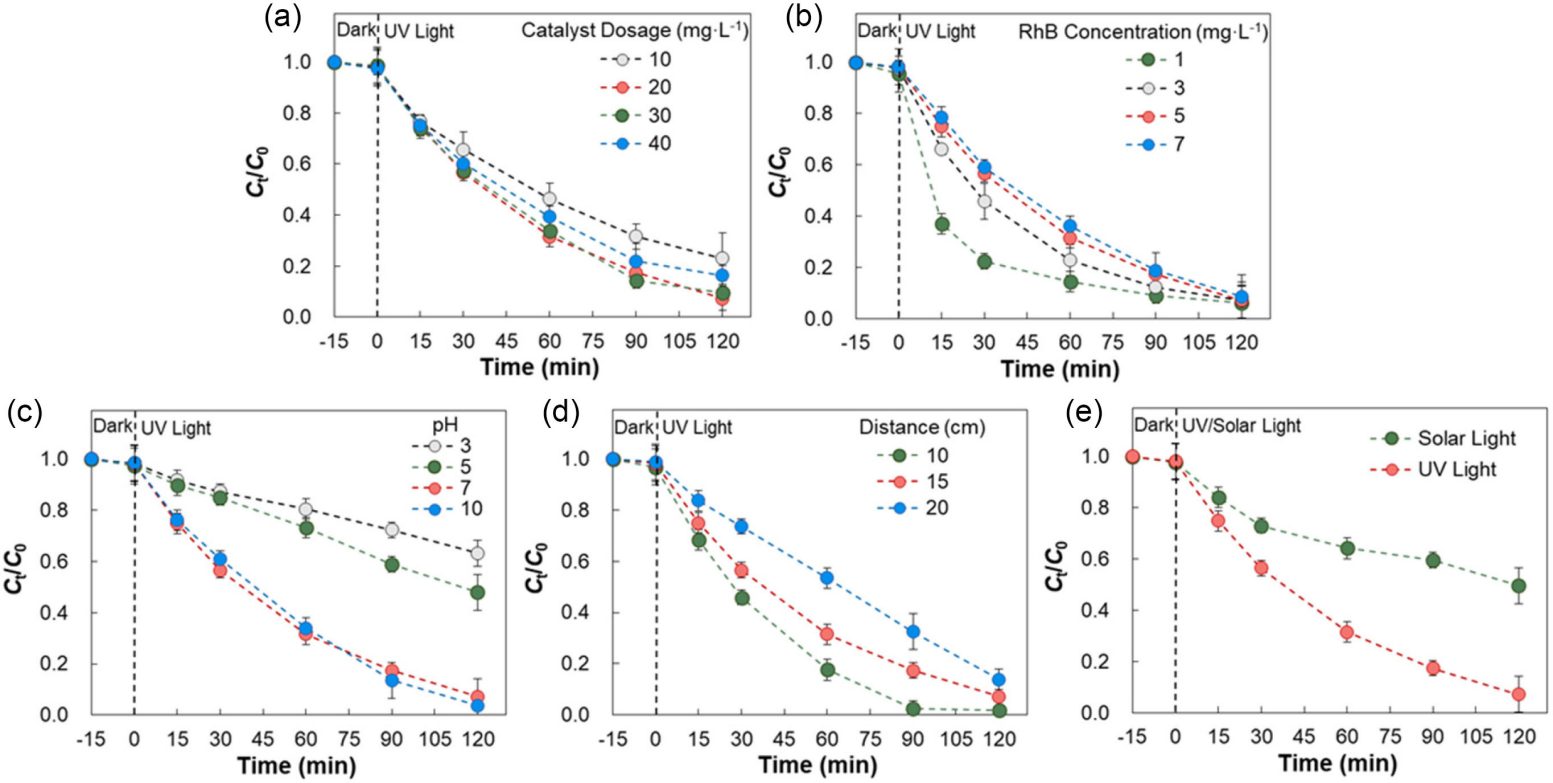
Effects of operating parameters on RhB degradation efficiency: (a) catalyst dosage, (b) initial RhB concentration, (c) pH, (d) irradiation distance, and (e) light source. Reaction conditions: unless otherwise stated, all photocatalytic experiments were conducted under the following conditions: RhB 5 mg/L, catalyst dosage 20 mg/L, irradiation distance 15 cm, LDE lamp (365 nm, 15w), pH 7.0, 25 °C. Error bars represent the standard deviation of 2 replicates.

Catalyst dosage plays an important role in photocatalytic degradation. To investigate its effect on RhB degradation, different dosages ranging from 10 to 40 mg·L^−1^ were used (Figure 4a). In the first half‐hour, the effect of catalyst dosage on photocatalytic performance was not significant. However, as the reaction progressed, increasing the catalyst dosage promoted RhB degradation to a certain extent. When the catalyst dosage was 20 mg·L^−1^, the RhB degradation percentage reached its maximum value of 92.7% after 120 min. Beyond this dosage, further increase in catalyst amount led to a decrease in RhB degradation. This can be attributed to the increased surface area of the photocatalyst available for adsorption and degradation as the dosage increased, but the reduced transparency of the reaction solution at higher dosages limited the amount of photogenerated reactive species, thus lowering the RhB degradation rate. Additionally, the deactivation of reactive molecules may occur due to collisions with nonreactive molecules [61, 63].

#### 3.5.2 Initial RhB Concentration

Photocatalytic oxidation experiments of RhB were conducted at various initial RhB concentrations. When the RhB concentrations were 1, 3, 5, and 7 mg·L^−1^, its photocatalytic degradation reached 98.8%, 95.8%, 92.8%, and 87.9% after 120 min, respectively (Figure 4b). As the initial concentration of RhB increased, its degradation rate gradually decreased. This decline is primarily due to three factors: first, as the concentration of RhB increased, more UV light was absorbed by the reactants in the solution, leaving fewer photons available for the catalyst to absorb. Second, the reactive sites on the photocatalyst are limited, and when the amount of RhB molecules exceeded the amount of available reactive sites, the proportion of degraded RhB decreased. Finally, at higher initial RhB concentrations, the concentration of generated intermediates also increased. These intermediates may compete with RhB for adsorption sites on the catalyst, and some intermediates may even revert to the parent RhB, further reducing the overall degradation efficiency [64, 65].

#### 3.5.3 Initial pH

Solution pH was adjusted to desired values using 0.1 M HCl or NaOH to investigate the effect of the catalyst on RhB degradation under pH conditions of 3.0, 5.0, 7.0, and 10.0. As shown in Figure 4c, the synthesized 5% CeO_2_/ZnO photocatalyst contributed to higher degradation of RhB under weakly alkaline conditions, with its catalytic activity decreasing as the pH value of the solution decreased. At a pH of 3.0, the degradation of RhB after 120 min was only 33.7%. This behavior can be explained by the molecular structure of RhB and the photocatalytic reaction mechanism. RhB molecules contain basic amine groups that undergo protonation under acidic conditions, which hindered the adsorption of RhB onto the surface of ZnO. Additionally, the formation of intermediate Zn(OH)_4_^2^^‒^ was inhibited in acidic environments. Since ZnO is an amphoteric oxide, its surface structure may be altered under acidic conditions, reducing its ability to adsorb and degrade RhB, thereby affecting its photocatalytic efficiency. On the contrary, under neutral or weakly alkaline conditions, the ZnO catalyst surface carries a negative charge, which facilitates the migration of h^+^ to the surface, where it can react with electron donors to produce HO^•^, thereby promoting photocatalytic reactions.

In addition to the adsorption effect, the decreased photocatalytic activity under acidic conditions can also be attributed to the chemical instability of ZnO. ZnO, as an amphoteric semiconductor, is susceptible to proton‐induced dissolution in acidic media according to: ZnO + 2H^+^ → Zn^2+^ + H_2_O. This process leads to partial surface corrosion, structural degradation, and loss of active sites, thereby weakening its photocatalytic performance. Such acid‐driven dissolution has been widely reported for ZnO‐based photocatalysts [66, 68].

#### 3.5.4 Irradiation Distance

The intensity of UV light is directly related to the photocatalytic degradation efficiency. This intensity can be adjusted by changing the distance between the UV lamp and the liquid surface of the reaction solution. As the irradiation distance increased, the degradation efficiency of RhB significantly decreased (Figure 4d). This indicates that lower UV light intensity leads to lower catalytic efficiency of the synthesized 5% CeO_2_/ZnO photocatalyst. The possible reason is that during the photocatalytic reaction, the activity of the photocatalyst increased with the generation of more photogenerated charges (including e^‐^ and h^+^).

#### 3.5.5 Light Source

Sunlight was employed as an additional irradiation source to evaluate the photocatalytic performance of the photocatalyst. The absorbance of the reaction solution was monitored at predetermined intervals to calculate RhB degradation. As shown in Figure 4e, the photocatalytic efficiency under 365 nm UV irradiation was higher than that under sunlight, achieving 92.8% RhB degradation after 120 min, compared with 50.4% under identical conditions. Despite this, the 5% CeO_2_/ZnO photocatalyst exhibited appreciable activity under sunlight, reaching approximately two‐thirds of the degradation efficiency observed under UV irradiation. From an economic standpoint, this performance indicates that 5% CeO_2_/ZnO is well‐suited for applications involving high light intensity and prolonged irradiation, where solar energy can be effectively utilized for organic pollutant removal, highlighting its promising practical potential and economic value.

### 3.6 Mechanism for Enhanced Degradation

**FIGURE 5:**
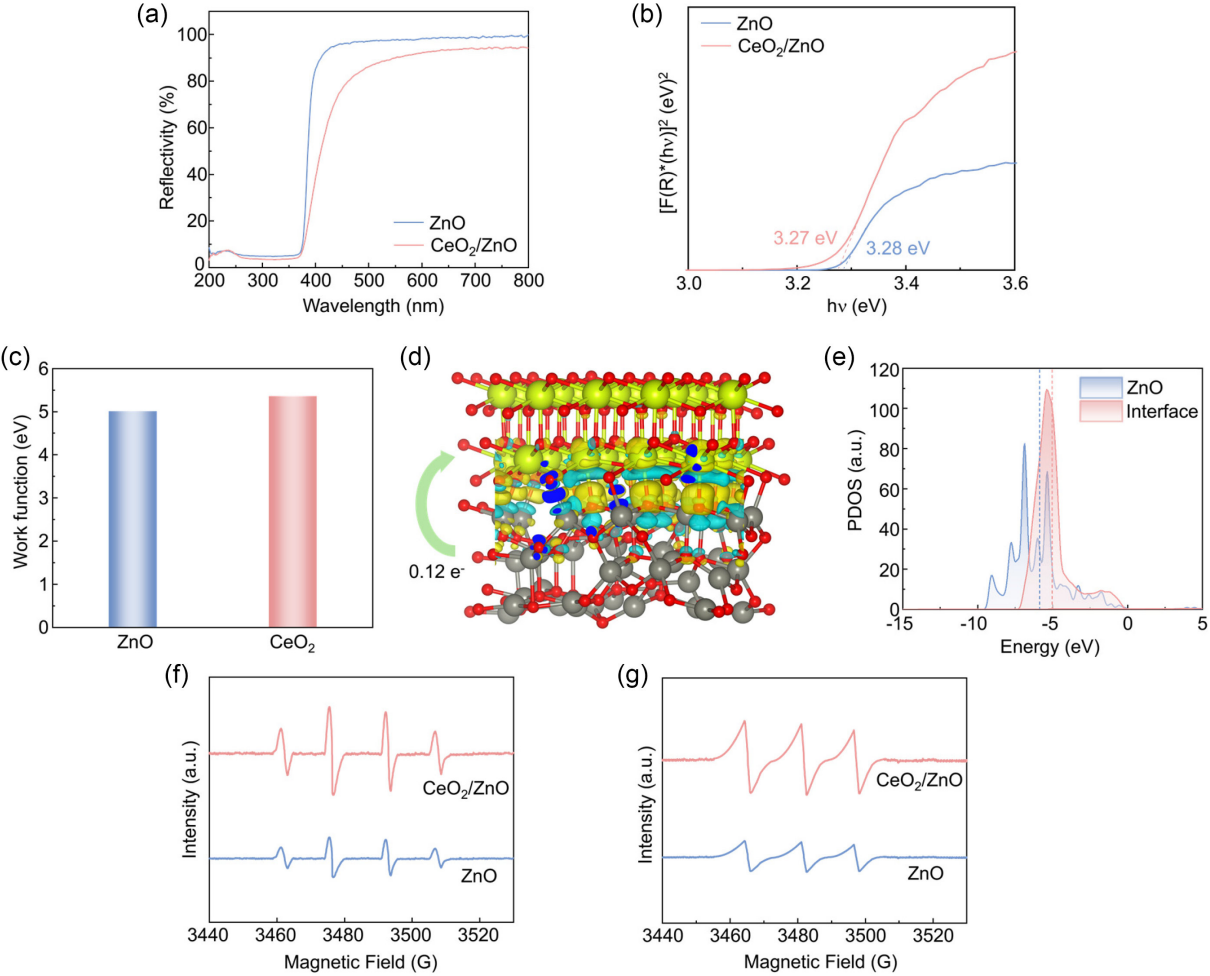
(a) UV–vis diffuse reflectance spectra (DRS) of ZnO‐S and 5% CeO_2_/ZnO‐S; (b) corresponding Tauc plots derived from the Kubelka–Munk function for optical bandgap estimation; (c) work function of ZnO and CeO_2_; (d) differential charge analysis indicating 0.12 e^‐^ was transferred from ZnO to CeO_2_; (e) PDOS of ZnO and ZnO–CeO_2_ interface; (f) EPR spectra of HO^•^; (g) EPR spectra of ^1^O_2_.

To experimentally verify the influence of CeO_2_ deposition on the optical properties of ZnO, UV–vis diffuse reflectance spectroscopy (DRS) was performed (Figure 5a). Compared with pristine ZnO, the CeO_2_/ZnO heterojunction exhibited a noticeably lower reflectance over the visible‐light region, indicating enhanced light harvesting after CeO_2_ deposition. The enhanced visible‐light absorption can be attributed to the introduction of CeO_2_, which modifies the interfacial optical response of the composite and increases the utilization of incident light. The optical bandgaps were further estimated from Tauc plots using the Kubelka–Munk function (Figure 5b). The calculated bandgap energies were 3.28 eV for ZnO‐S and 3.27 eV for 5% CeO_2_/ZnO‐S. The negligible difference indicates that CeO_2_ deposition does not substantially alter the intrinsic bandgap of ZnO. Therefore, the enhanced photocatalytic performance cannot be primarily attributed to bandgap narrowing.

Since the optical bandgap remained nearly unchanged, we further examined whether the enhanced activity originated from interfacial electronic interactions between CeO_2_ and ZnO. DFT calculations were employed to elucidate the electronic interactions at the CeO_2_/ZnO interface. The work function of ZnO (5.01 eV) is lower than that of CeO_2_ (5.36 eV), indicating its weaker electron‐holding capacity and stronger tendency to donate electrons (Figure 5c). Upon heterojunction formation, spontaneous interfacial electron transfer (0.12 e) occurs from ZnO to CeO_2_, as revealed by differential charge analysis (Figure 5d). This electron redistribution, primarily mediated through the Zn–O–Ce bridge, induces a positive shift of Zn d‐band center (from −5.87 to −5.02 eV) toward the Fermi level (Figure 5e), signifying enhanced surface reactivity. According to the d‐band center theory, this upshift enhances the overlap between Zn d states and the antibonding π* orbitals of molecular oxygen [69], thereby strengthening the adsorption interaction and lowering the adsorption free energy of O_2_. Thermodynamically, this results in a more stable O_2_ adsorption configuration and facilitates interfacial electron transfer from the catalyst surface to adsorbed oxygen species, effectively weakening the O=O bond [70, 71]. Consequently, the activated O_2_ molecules are more readily converted into ROS, contributing to the enhanced RhB degradation.

EPR further confirmed the ROS generated on the interface during photocatalysis. EPR measurements were conducted using 5,5‐dimethyl‐1‐pyrroline‐N‐oxide (DMPO) and 2,2,6,6‐tetramethyl‐4‐piperidinol (TEMP) as spin trapping agents to detect hydroxyl radical (HO^•^) and singlet oxygen radical (^1^O_2_), respectively. The characteristic DMPO‐HO^•^ (Figure 5f) and TEMP‐^1^O_2_ (Figure 5g) signals were clearly observed under UV irradiation for both ZnO and CeO_2_/ZnO catalysts, indicating the formation of HO^•^ and ^1^O_2_. Notably, the CeO_2_/ZnO catalyst exhibited significantly enhanced EPR signal intensities for both HO^•^ and ^1^O_2_ relative to ZnO, implying more efficient ROS generation upon Ce incorporation. These results explain the higher degradation rate of RhB in the CeO_2_/ZnO photocatalytic process.

It is well established that the photocatalytic activity of semiconductor materials originates from photoexcitation, whereby absorption of photons with energies equal to or exceeding the bandgap leads to the generation of e^‒^/h^+^. The photogenerated charge carriers subsequently migrate to the catalyst surface and participate in redox reactions with adsorbed oxygen‐containing species, resulting in the formation of ROS. For ZnO‐based photocatalysts, HO^•^ is typically the dominant ROS due to the strong oxidative ability of valence band (VB) holes [72, 73]. Upon UV excitation, these holes readily oxidize surface‐adsorbed H_2_O or OH^‐^ to produce HO^•^, which accounts for the pronounced DMPO‐HO^•^ signals observed in the EPR spectra. In contrast, ^1^O_2_ generally plays a subsidiary role in such systems; however, its formation is not uncommon and may originate directly from defects in nanocrystalline ZnO [74], from the disproportionation of superoxide radicals (O_2_^•^^‒^) or from energy‐transfer processes involving photoexcited RhB molecules. In the CeO_2_/ZnO heterostructure, the formation of a heterojunction enhances the separation and lifetime of photogenerated charge carriers, enabling more electrons and holes to participate in surface reactions and thereby increasing the overall ROS yield. Meanwhile, surface defects and the reversible Ce^3+^/Ce^4+^ redox couple associated with CeO_2_ facilitate oxygen activation and electron/energy transfer, moderately promoting O_2_‐related pathways, including ^1^O_2_ generation. The synergistic enhancement of ROS production‐dominated by hydroxyl radicals‐ultimately accounts for the superior photocatalytic degradation performance of RhB over the CeO_2_/ZnO catalyst.

**FIGURE 6:**
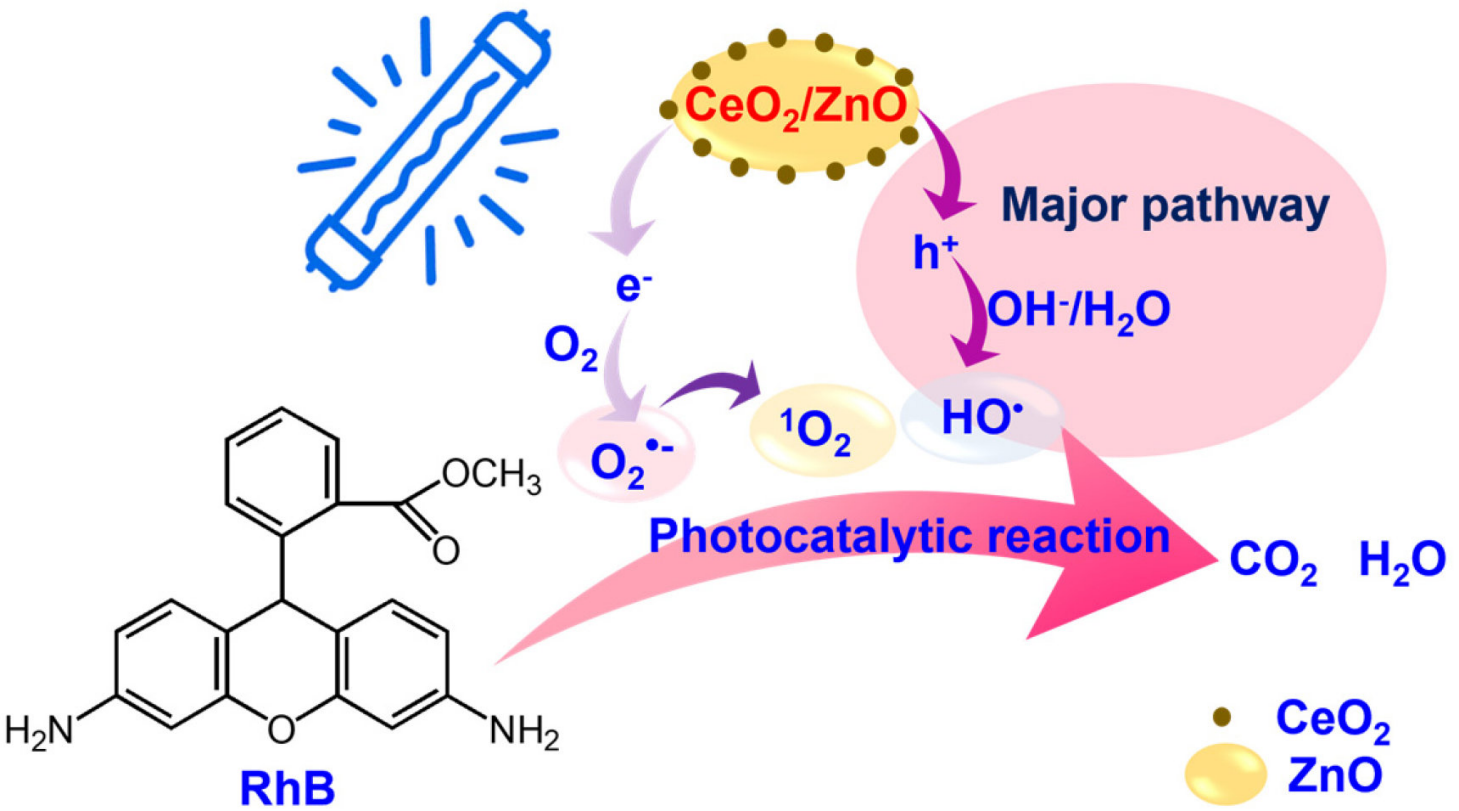
Reaction mechanism of CeO_2_/ZnO photocatalytic for RhB degradation.

It should be noted that DMPO is capable of trapping both HO^•^ and O_2_^•^^‒^. However, no formation of O_2_^•^^‒^ was detected either with or without Ce incorporation. This absence does not necessarily exclude the formation of O_2_^•^^‒^ during the photocatalytic process. In aqueous systems, DMPO reacts with HO^•^ to form the DMPO‐HO^•^ adduct, which is highly stable and gives rise to well‐defined quartet signals (1:2:2:1) [75, 76]. Although DMPO can also trap O_2_^•^^‒^ to generate the DMPO‐O_2_^•^^‒^ adduct, this species is intrinsically less stable and is prone to rapid decomposition or transformation. In particular, DMPO‐O_2_^•^^‒^ can readily undergo protonation and subsequent conversion into DMPO‐HO^•^ in water, resulting in the dominance of DMPO‐HO^•^ signals and the suppression or disappearance of detectable DMPO‐O_2_^•^^‒^ features [76, 77]. Several studies have reported the concurrent detection of multiple ROS during ZnO photocatalysis, including HO^•^, O_2_^•^^‒^, and ^1^O_2_; however, the contribution of O_2_^•^^‒^ to RhB degradation is often limited relative to that of HO^•^ and VB holes [78]. Therefore, the possible mechanism is depicted in Figure 6. Under UV irradiation, ZnO absorbs photons with energy higher than its bandgap, resulting in the excitation of electrons from the VB to the conduction band (CB), and the generation of photogenerated holes in the VB [49, 79]:

(5) $$\mathrm{ZnO}+h\text{ν}\to e_{\mathrm{CB}}^{-}+h_{\mathrm{CB}}^{+}$$

The photogenerated electrons can react with dissolved oxygen molecules adsorbed on the catalyst surface to generate superoxide radicals:

(6) $$e_{\mathrm{CB}}^{-}+O_{2}\to O_{2}^{•\text{−}}$$

Meanwhile, photogenerated holes oxidize surface hydroxyl groups or H_2_O to generate HO^•^:

(7) $$h_{\mathrm{CB}}^{+}+H_{2}O/{\mathrm{OH}}^{-}\to {\mathrm{HO}}^{•}$$

Furthermore, the interaction between photogenerated charges and oxygen‐containing species facilitates the formation of ^1^O_2_. The incorporation of CeO_2_ enhances the photocatalytic activity of the CeO_2_/ZnO heterostructure by promoting interfacial charge separation through the Ce^3+^/Ce^4+^ redox cycling and oxygen vacancy‐mediated electron transfer. The oxygen vacancies in CeO_2_ serve as electron trapping sites, facilitating the migration of photogenerated electrons and suppressing e^‒^/h^+^ recombination. As a result, the increased availability of charge carriers participates in surface redox reactions, thereby promoting the generation of ROS. This enhanced ROS formation is evidenced by the stronger characteristic EPR signals observed for the CeO_2_/ZnO heterostructure compared with pristine ZnO, confirming the improved oxygen activation capability induced by CeO_2_ incorporation. Such enhancement can be attributed to the synergistic effects of heterojunction‐induced charge separation and the Ce^3+^/Ce^4+^ redox couples and oxygen vacancies, which facilitate electron/energy transfer processes, consistent with the DFT calculations and band structure analysis. The increased availability of HO^•^, which reacts with RhB at significantly higher rates than O_2_^•^^‒^ due to its higher oxidation potential, provides a mechanistic basis for the observed enhancement in photocatalytic RhB degradation over the CeO_2_/ZnO catalyst [80].

### 3.7 Recyclability of the Photocatalysts

**FIGURE 7:**
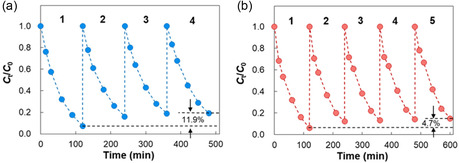
Recyclability property of (a) 5% CeO_2_/ZnO and (b) magnetic M‐CeO_2_/ZnO.

From an economic perspective, the recoverability and reusability of a photocatalyst are critical [81]. After centrifugation and drying, the 5% CeO_2_/ZnO photocatalyst was evaluated over repeated RhB degradation cycles. As shown in Figure 7a, the RhB degradation efficiency decreased by 11.9% after four cycles, demonstrating good reusability. Notably, the degradation efficiency decreased more markedly in the second cycle relative to the preceding cycle, whereas only minor declines were observed in the third and fourth cycles. This gradual decline can be attributed to inevitable photocorrosion of the photocatalyst, as reported previously [82, 83], as well as active site coverage and pore blockage. Additionally, reaction intermediates may adsorb onto photocatalyst active sites, competing with RhB for adsorption and thereby inhibiting its degradation. Catalyst loss during the recovery process may further contribute to the observed decrease in degradation efficiency, consequently affecting the measured catalytic performance. After cycling experiments, the reaction suspension was filtered, and the filtrate was analyzed to determine the dissolved Ce concentration. The Ce concentration in the solution was below the detection limit of ICP‐OES, indicating negligible Ce leaching during photocatalytic degradation.

Given the complexity of centrifugation‐based catalyst recovery, a magnetic photocatalyst was developed by incorporating 10% ZnFe_2_O_4_ to enhance recoverability while maintaining catalytic performance. The resulting magnetic property enables facile manipulation by an external magnetic field, allowing repeated reuse. As shown in Figure 7b, the RhB degradation efficiency decreased by only 4.7% after four cycles and remained nearly constant after three cycles, demonstrating excellent recyclability. Consequently, M‐CeO_2_/ZnO is a promising recyclable photocatalyst. Furthermore, ZnFe_2_O_4_ was introduced not only as a magnetic component for catalyst recovery but also as an active semiconductor component to construct a structurally compatible heterojunction. Owing to its stable spinel oxide framework and abundant metal–oxygen coordination sites, ZnFe_2_O_4_ can form intimate interfacial contact with CeO_2_/ZnO, which helps preserve the structural integrity of the composite during repeated photocatalytic cycles [84, 85].

In addition, the narrow bandgap of ZnFe_2_O_4_ (~1.9 eV) enables photoinduced charge transfer and extends light utilization [86]. The ZnFe_2_O_4_/CeO_2_/ZnO interface promotes spatial separation of photogenerated electron–hole pairs, suppresses charge recombination, and alleviates photocorrosion, thereby contributing to the enhanced stability and recyclability of M‐CeO_2_/ZnO [87]. Therefore, the improved reusability of M‐CeO_2_/ZnO results from the combined effects of magnetic separation, structural compatibility, and interfacial charge–transfer regulation.

## 4 Conclusion

In summary, we have demonstrated a systematic approach to enhance the photocatalytic performance of ZnO by integrating morphology control, interfacial engineering, and functional composite design. Sphere‐like ZnO was identified as the optimal support due to its favorable structural and surface properties. The formation of a CeO_2_/ZnO heterojunction introduced mixed‐valence cerium species and induced interfacial charge transfer, effectively suppressing charge recombination and promoting ROS generation. Both experimental characterizations and theoretical calculations revealed that CeO_2_ deposition modulates the electronic structure of ZnO by shifting the Zn d‐band center toward the Fermi level, thereby enhancing surface reactivity toward oxygen activation. Furthermore, the incorporation of ZnFe_2_O_4_ endowed the catalyst with magnetic separability and improved cycling stability, addressing a key limitation for practical application. These findings highlight the importance of coupling structural design with electronic modulation in oxide photocatalysts and provide a general strategy for developing efficient and recyclable photocatalytic systems for environmental remediation. Future studies are also expected to extend the evaluation to a wider range of organic pollutants with different chemical structures, to further demonstrate the versatility of the catalyst system.

## Author Contributions

**Bingwei Zhong**: investigation, methodology, writing – original draft, funding acquisition. **Bo Xia**: software, formal analysis. **Nan Wang**: methodology. **Jing Hu**: methodology, writing – review and editing. **Xulei Zhao**: conceptualization, writing – review and editing, supervision, funding acquisition.

## Funding

This work was supported by the Department of Education of Zhejiang Province (Y201942497), Chinese Academy of Sciences (XDA09030103) and Research Foundation of Jiyang College of Zhejiang A&F University (RC2024F03).

## Conflicts of Interest

The authors declare no conflicts of interest.

## Supporting information

Supplementary Material

## Data Availability

Data will be made available on request.
