# Supplementary material for: Boosting Photocatalytic Performance of ZnO via Ce‐Induced d‐Band Modulation: Synthesis and Rhodamine B Degradation
Source: ChemistryOpen. 2026 Jul 15;15(7):e70260. doi: 10.1002/open.70260 (PMC13373456; doi:10.1002/open.70260)
Supplement: Supplementary file 1 — Supplementary Material [file OPEN-15-e70260-s001.pdf]

Supporting Information

Boosting photocatalytic performance of ZnO via Ce-induced d-band modulation: synthesis and rhodamine B degradation

Bingwei Zhong[1], Bo Xia[1], Nan Wang[1], Jing Hu[2*], Xulei Zhao[1*]

[1]College of Jiyang, Zhejiang A&F University, Zhuji 311800, China

[2]School of Earth, Atmosphere and Environment, Monash University, Clayton, VIC 3800, Australia

*Corresponding authors: xuleizhao@zafu.edu.cn; jing.hu1@monash.edu

2 Figures

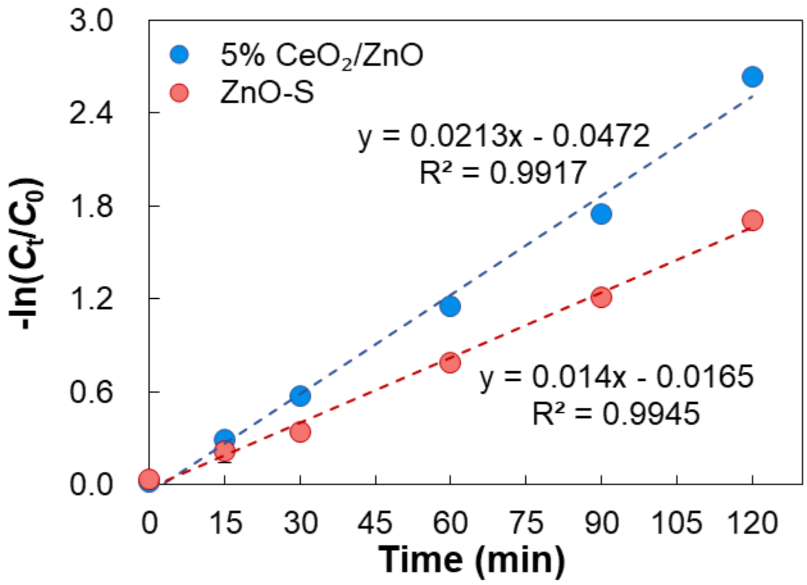

Figure S1. Linear plots for RhB degradation under catalytic conditions.

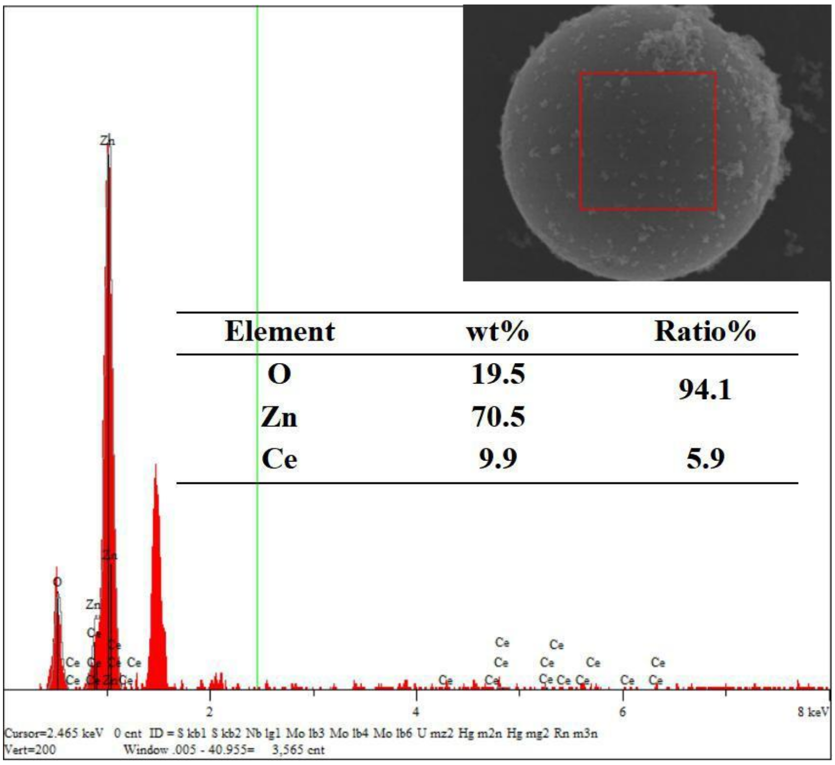

Element	wt%	Ratio%
O	19.5	94.1
Zn	70.5	
Ce	9.9	5.9

Figure S2. EDS spectra of 5% Ce-loaded sphere-like ZnO.

Table S1 ICP analysis for Ce leaching

Ce loading	1%	3%	5%	7%
Dissolved Ce ions	ND	ND	ND	ND

Text S1

The incident photon flux (I_0) of the UV irradiation source was determined by chemical actinometry using a p-nitroaniline (PNA)/pyridine (pyr) system [1]. Briefly, a mixed solution containing 7.9 μM PNA and 1.24 mM pyr was irradiated under the same experimental conditions as the photocatalytic reaction using a 365 nm UV lamp. The photodegradation kinetics of PNA were monitored by measuring the concentration change of PNA as a function of irradiation time. The observed degradation rate constant (k_{obs}) was obtained according to the pseudo-first-order kinetic equation:

$$\ln(\mathrm{C_t}/\mathrm{C_0}) = -k_{\mathrm{obs}}\cdot\mathrm{t} \qquad \text{eq. S1}$$

where $\mathrm{C_0}$ and $\mathrm{C_t}$ represent the initial concentration of PNA and the concentration at irradiation time (t), respectively. The incident photon flux was calculated using the following equation:

$$k_{\mathrm{obs}}\cdot\mathrm{C_0} = I_0[1 - 10^{-(\varepsilon_{PNA}c_{PNA})l}]\Phi_{PNA} \qquad \text{eq. S2}$$

where (I_0) is the incident photon flux, ε_{PNA} (2276 $M^{-1}cm^{-1}$) is the molar extinction coefficient, l is the optical path length (6.8 cm), and Φ_{PNA} (9.0×10^{-4} M Einstein $^{-1}$) is the quantum yield of PNA photodegradation at 365 nm. Based on the measured k_{obs} (4.235×10^{-4} s^{-1}), the incident photon flux of the UV lamp at 365 nm was calculated to be 9.0 ×10^{-8} Einstein·cm^{-2}·s^{-1}.

Reference:

[1] D. Dulin, T. Mill, Development and evaluation of sunlight actinometers, Environ. Sci. Technol. 16 (1982) 815-820.
